# A large cluster randomized trial of outcome-based pathways to improve home-based wound care

**DOI:** 10.1186/s13063-017-2082-5

**Published:** 2017-08-29

**Authors:** Merrick Zwarenstein, Salimah Shariff, Nicole Mittmann, Anita Stern, Katie N. Dainty

**Affiliations:** 1 Centre for Studies in Family Medicine, Department of Family Medicine, Schulich School of Medicine and Dentistry, Western University, 1151 Richmond St, London, N6A 3K7 ON Canada; 2 TAGlab (Technologies for Aging Gracefully lab.), University of Toronto, rm 7214, 40 St. George St, Toronto, ON M5S 2E4 Canada; 3 Institute for Clinical Evaluative Sciences, Western Satellite Site, London, ON Canada; 4 HOPE Research Centre, Sunnybrook Health Sciences Centre, Toronto, ON Canada; 5 Li Ka Shing Knowledge Institute, St. Michael’s Hospital, Toronto, ON Canada; 6 Institute of Health Policy Management and Evaluation, University of Toronto, Toronto, ON Canada

## Abstract

**Background:**

Although not always recognized as a pressing health care problem, wounds are a common, complex and costly condition. The burden of treating wounds is growing rapidly due to increasing health care costs, an aging population and a sharp rise in the incidence of diabetes and obesity worldwide. The Integrated Client Care (ICC) Project was a multi-year initiative to develop and test a new, integrated model of wound care within the home care sector in Ontario, Canada to improve health outcomes for patients and decrease system costs.

**Methods:**

Cluster randomized trial, with allocation of intervention randomized at the cluster level (14 home care centers) and analysis of outcomes based on individual-level data (patients). Primary analysis was an intention-to-treat (ITT) analysis. Two wound types, diabetic foot ulcers and pilonidal sinus, were selected as tracer conditions to assess the impact of the intervention on two different patient populations. Time to successful discharge from home care was analyzed using multivariable Cox proportional hazards regression. Hazard ratios (HRs) and 95% confidence intervals (CIs) are presented.

**Results:**

A total of 12,063 diabetic foot ulcer patients and 1954 pilonidal sinus patient records were available for analysis. No appreciable differences were observed between patients in the control and intervention arms for either of the primary or secondary analyses in either condition group. In the diabetic foot ulcer group, 72.7% patients in the control arm and 73.6% patients in the intervention arm were discharged in the follow-up period (HR 1.05; 95% CI 0.94 to 1.17). In the pilonidal sinus group, 91.0% patients in the control arm and 89.0% patients in the intervention arm were discharged in the follow-up period (HR 0.96; 95% CI 0.82 to 1.12).

**Conclusion:**

As implemented, the ICC intervention was not effective, most likely due to failure of implementation, and is, therefore, not ready for widespread implementation in Ontario. Significant work remains to be done to correct the implementation process so that the concept of outcome-based health care can be properly evaluated.

**Trial registration:**

ClinicalTrials.gov, ID: NCT01573832. Registered on 12 January 2012.

## Background

Although not always recognized as a pressing health care problem, wounds are a common, complex and costly condition [1]. Approximately 1.5 million Ontarians will sustain a pressure ulcer, 111,000 will develop a diabetic foot ulcer and between 80,000 and 130,000 will develop a venous leg ulcer [1]. Up to 65% of patients afflicted by chronic wounds report experiencing decreased quality of life, restricted mobility, anxiety, depression and/or severe or continuous pain [2]. The estimated cost to care for a pressure ulcer in the community was CDN$27,000 in 2006, and has risen in the last decade [3].

The burden of treating wounds is growing rapidly due to increasing health care costs, an aging population and a sharp rise in the incidence of diabetes and obesity worldwide [4]. Community-based care for people with wounds is often fragmented and inconsistent, leading to prolonged healing times and ineffective use of resources. Appropriate clinical management of wounds requires a team-based, coordinated approach to diagnosis, treatment and patient education [5]. In May 2009, our group was selected through a peer-review process to conduct an independent, prospective evaluation of the implementation and impact of an integrated model of wound care within the home care sector in Ontario, Canada. The evaluation consisted of a mixed-methods, realist approach. This report details the quantitative evaluation of a pragmatic cluster randomized trial for the management of wounds in 14 home care centers.

## Methods

### Setting

Ontario, Canada is the most populous Canadian province with approximately 13.6 million residents (2014 estimate), representing 40% of the Canadian population (http://www.fin.gov.on.ca/en/economy/ecupdates/factsheet.html). Residents are covered by a single-payer, universal health insurance program that includes almost all physician services and inpatient care, as well as most home care and some long-term care. Fourteen geographically organized, publicly funded Community Care Access Centers (CCACs) have the responsibility for the coordination of the majority of home care services (including rehabilitation, post-acute care and medically required domestic help), for all Ontarians requiring these services (over 700,000 referrals in 2013/2014) [6]. CCACs do so through complex bulk service contracts with a range of large and small, for-profit and not-for-profit, multidisciplinary and unidisciplinary private Service Provider Organizations (SPOs). Patients are referred to CCAC case managers by their primary-care physician at discharge from hospital care or through other community-based health providers. A CCAC case manager assesses each patient’s home care needs, including for wound care, and, if they are in need of care, enters them into a referral database that allocates the patient to one of the partner SPOs according to a market-based randomization algorithm. Home care is provided on either a short- or long-stay basis, where the latter refers to patients who are anticipated to receive care for a minimum of 60 days in a single episode [7].

The specific care to be provided is determined by the SPO employee (nurse, physiotherapist, occupational therapist, etc.) and is approved by a case manager from the CCAC. The specific services and the intensity of services provided vary depending on client need, but may consist of homemaking, personal support (i.e., assistance with bathing), and health professional visits (i.e., nursing, physiotherapy). Since the system is publicly funded, service maximums have been put in place; for example, homemaking services cannot exceed 60 hours in a 30-day period. Currently, this is reimbursed on a fee-for-service basis, with the fee determined in negotiations between SPOs and CCACs every few years. The system is currently noncompetitive, with market share for each SPO fixed by contract.

### The intervention

As the steward of publicly funded health care in Ontario, the Ministry of Health and Long-term Care (MOHLTC) began seeking an organizational and structural redesign that would both improve quality of care and bend the curve of rising system costs for wound care. The 2005 study by Harrison et al. showed that remedying these issues could improve the effectiveness and efficiency of delivery and of outcomes of wound care [1] and the MOHLTC objective was to implement a tailored intervention across the province in a sustainable fashion.

**Fig. 1:**
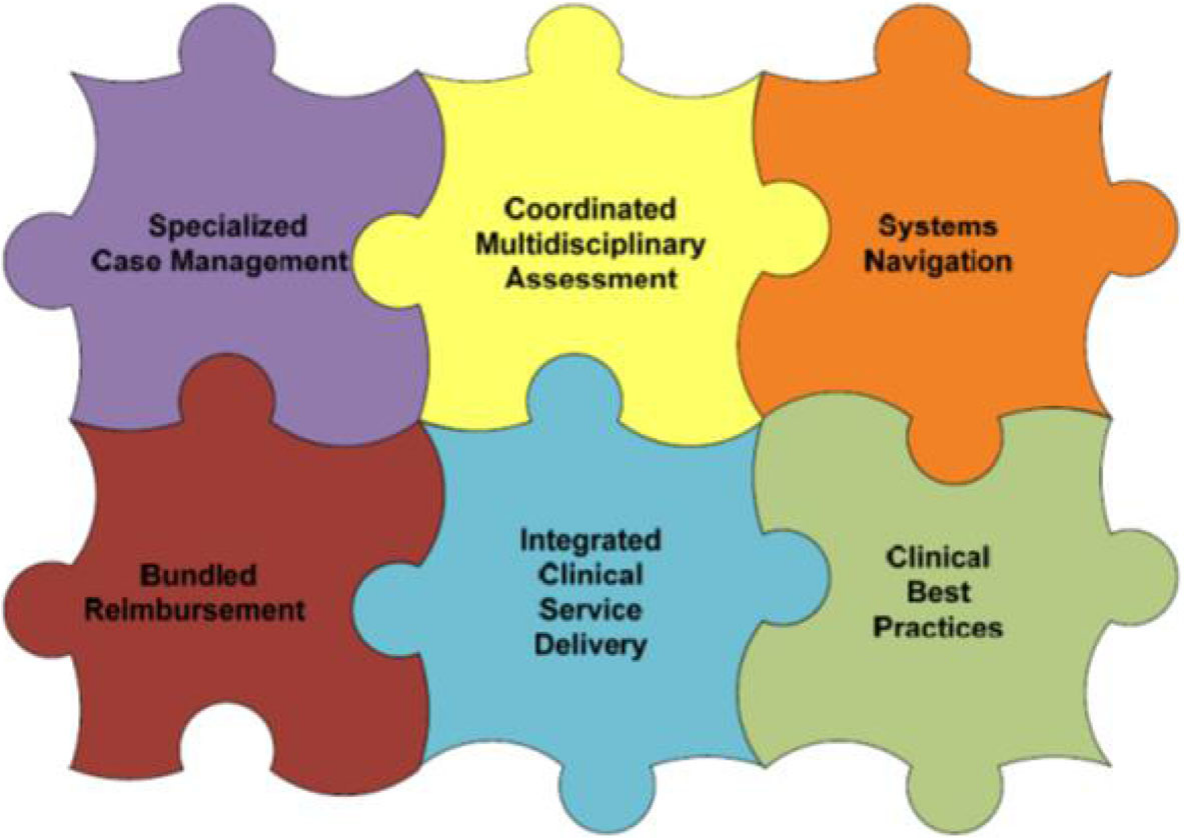
Visual depiction of the six elements in the theoretical Integrated Client Care (ICCP) model of care

The Integrated Client Care (ICC) Project began as a multi-year initiative to develop and test a new, integrated model of home care in Ontario which would improve health outcomes for patients without substantially increasing system cost. This was to be achieved by more accurate matching of care provided to each specific patient’s needs. At system level, this was expected to redirect care resources towards more complex patients. The ICCP Project was designed to be an innovation development and demonstration collaboration between the payer (MOHLTC) and the sector (CCACs and SPOs) and was sponsored and funded by the MOHLTC. Because of the key coordinating role of the CCACs, the MOHLTC commissioned their representative body, the Ontario Association of CCACs (OACCAC) to develop and implement the new model of care. The model of care was to be implemented using six strategies (Fig. 1) and the first clinical grouping chosen on which to test ICCP was community-based wound care.

### Study design

In parallel with a longitudinal qualitative evaluation, the quantitative evaluation reported here was designed as a cluster randomized trial, with allocation of intervention randomized at the cluster level (CCAC) and analysis of outcomes based on individual-level data (patients). Comparisons were made with clusters allocated to usual care (control arm).

In this work we were evaluating an intervention on behalf of policy-makers, assessing its effectiveness and prospects for province-wide scale up. This was particularly suitable for a pragmatic approach to a randomized controlled trial (pRCT), which is a specific way of designing a randomized trial to give information on “real-world” effectiveness, evaluating outcomes under conditions of actual use. Because of this, pRCTs can yield results that are both valid and generalizable, and thus relevant for making real-world decisions about changes in practice guidelines, formularies or payment structures [8, 9].

### Intervention and comparison groups

The new model of care was planned to be implemented through six integrated changes to home care organization, delivery and reimbursement to promote improved outcomes and integrated care (Fig. 1).

Under the leadership of the OACCAC, the sector developed 10 best-practice outcome-based wound care pathways and two pathways for rehabilitation after hip or knee replacement (Table 1). Milestone-based outcome^1^ pathways were developed via an enhanced portal within the Client Health and Related Information System (CHRIS). CHRIS is a web-based patient management system used by all Ontario CCACs to access patient information and care plan details in real time. For each wound condition, CCACs randomized to intervention had access to the electronic pathway documentation; those CCACs randomized to usual care continued to use CHRIS as per usual business. In the interventional group, progress of each patient along these pathways was entered directly into the CHRIS electronic record by front-line clinicians, or on paper and then entered later by office staff. The control CCACs were not intended to undergo any changes in delivery, organization or reimbursement and were expected to continue with usual care and usual reporting of wound healing.

**Table 1:** List of wounds targeted by outcome-based pathways

Arterial leg ulcer	Pilonidal sinus
Diabetic foot ulcer	Pressure ulcer
Maintenance wound	Surgical wound
Malignant wound	Traumatic wound
Nonhealing wound	Venous leg ulcer

Among the interventional group, the appropriate pathway for each patient was determined initially through a CCAC case manager assessment (without direct contact with the patient, based on referral information) and confirmed by the first visiting front-line clinician, usually a nurse, employed by a SPO. Patient progress through the milestones specific to their particular wound and general health needs was recorded by front-line clinicians, and also monitored by CCAC case managers with the intention that they would intervene if progress was insufficient, or if required care was not provided. These outcomes pathways were developed by OACCAC and CCAC staff from the underlying clinical pathways^2^ for each wound care type (see attached outcome-based pathways for pilonidal sinus and diabetes lower limb ulcers – (Diabetic lower limb ulcer pathway: http://healthcareathome.ca/serviceproviders/en/Documents/DiabeticFootUlcer_V1.pdf Pilonidal sinus pathway: http://healthcareathome.ca/serviceproviders/en/Documents/PilonidalSinus_V1.pdf).

### Outcome measures: selection of tracer conditions

Collectively, the pathways classify all admitted patients’ wounds into one or more of 10 categories as listed in Table 1. We selected two of these wound types, diabetic foot ulcers and pilonidal sinus, as tracer conditions to assess the impact of the intervention on two different patient populations. Diabetic foot ulcers afflict older, sicker individuals and, if not managed successfully, may result in foot amputations and significant morbidity. This combination of diabetes and lower limb ulceration is very disabling and costly to the health care system [10]. Pilonidal sinus, on the other hand, is a condition that occurs mostly in younger, healthy adults, but can be difficult to heal due to the peri-anal location of the wound. It may result in significant personal economic impact due to lost work days [11].

### Study participants – clusters

Ontario is divided into 14 health regions, known as Local Health Integration Networks (LHINs) each with a Community Care Access Center (CCAC) that coordinates delivery of provincially funded community-based services. These 14 clusters in the province were randomized either to the arm which was supposed to receive ICCP wound care intervention (seven CCACs) or to the one allocated to usual care (seven CCACs). Randomization was stratified for size (larger and smaller) and geographic area (urban versus rural). Randomization occurred before the intervention was made available within the IT systems of the CCACs to avoid selection bias (July 2012). Once the randomization was determined, the intervention was immediately made available to the seven CCACs randomized to receive the intervention early. Due to the pragmatic nature of the trial, the CCACs had control over when they chose to begin using the intervention (this ranged from 5 to 12 months). During this time one of the control group CCACs requested, and was permitted to have access to, the intervention, and two of the intervention group CCACs declined to implement the intervention.

### Study participants – individual patients

The patient grouping used for analysis included all home care referrals admitted from 1 January to 31 December 2013 with a documented diagnosis for diabetic foot ulcer or pilonidal sinus (see definitions below) in the province of Ontario. The year 2013 was selected as the majority of CCACs that had applied the intervention had commenced the use of outcome-based pathways early that year.

### Data sources

In addition to the CHRIS, we used multiple, linked, population-based databases, including the Registered Persons Database (RPDB) which includes demographic information on all Ontario residents; the Canadian Institute for Health Information Discharge Abstract Database (DAD) which consists of standardized chart abstractions for all inpatient hospital episodes; the National Ambulatory Care Reporting System (NACRS) which consists of standardized reporting on all emergency department visits; the Ontario Health Insurance Plan (OHIP) which includes all billing claims for physicians paid on a fee-for-service basis, and the Home Care Database (HCD) which includes information about each patient, their primary diagnosis and services received for each referral. By using several linked databases we were able to include all of the information about any interactions that the study participants had across the health care system.

### Defining eligible referrals and follow-up periods

For analysis we developed two wound cohorts, one to represent more complex wounds in an older population (diabetic ulcers), and one to represent simpler wounds in a younger population (pilonidal sinus). We had two considerations in selecting the wound types for analysis. First, we required that the wounds have good specificity and minimal misclassification in administrative coding (i.e., a physician or coder would be confident when documenting the condition). Second, analysis required that the wound could be accurately identified using physician billings. The latter constraint was due the availability of hospital data; at the time of the analysis hospital discharge records were only available covering the period up to March 2013. As the majority of the referrals applying the pathways were admitted in 2013, using the available hospital data would have limited the timeframe for this analysis. However, physician billings were available throughout the period. For this tracer analysis, we thus selected pilonidal sinus and diabetic foot ulcer for further assessment as both conditions can be treated or clinically managed on an outpatient basis and are associated with specific billing or diagnostic coding.

For both cohorts we applied the same initial inclusion and exclusion criteria, as follows: we first included all home care referrals admitted between January and December 2013. We then excluded referrals where clients had invalid or incomplete data (missing age or sex, died prior to admission date), were under 18 year of age and where no nursing services were provided (i.e., only homemaking services were provided). For all analyses we commenced client follow-up from the referral admission date; clients were followed to a maximum follow-up date of 30 April 2014, allowing for a minimum of 4 months of follow-up for all clients.

We defined a referral as including a diagnosis of pilonidal sinus if a physician had billed for the treatment of the condition in the 30 days prior to the home care admission date. Similarly, for diabetic foot ulcers we required a diagnosis for diabetes, determined using a validated algorithm [12], and a physician-documented diagnosis for diabetic foot ulcer in the 30 days prior to home care admission. Billing and diagnostic codes used for case identifications are presented in Table 2. Given the severity of both conditions, we assumed that any subsequent home care requiring nursing services would have received some management for these diseases.

**Table 2:** Billing codes and descriptions used to identify cases

^a^Diagnostic and Billing codes	Description
707 with 250	Skin ulcer, bed sore, including Diabetes related ulcer
R035	Pilonidal cyst – simple excision or marsupialization
R054	Pilonidal cyst – simple excision or marsupialization if patient’s BMI >40
R036	Pilonidal cyst – excision and skin shift
Z106	Abscess or hematoma – local anesthetic – ischiorectal or pilonidal
Z107	Abscess or hematoma – general anesthetic – ischiorectal or pilonidal

^a^Physician diagnostic codes are restricted to the first three characters of the *International Classification of Diseases 9th Revision*

### Analysis

For each wound cohort we performed two analyses. Our primary analysis was an intention-to-treat (ITT) analysis, where arms were compared by intended allocation status (seven CCACs allocated to the intervention versus seven CCACs allocated to usual care). Our secondary analysis compared centers that applied pathways for the conditions of interest (pilonidal sinus or diabetic foot ulcer) to the remaining centers that did not (five intervention centers versus nine control centers); we refer to this as the “per-protocol analysis” (PP).

### Primary outcome

The primary outcome was defined as a successful discharge from home care, indicated as “service plan complete” in the HCD. We defined a discharge date as the date of last clinical service. We preferentially selected this date as clients could have continued to receive nonclinical services prior to formal discharge from home care.

### Additional variables

For each client, we collected baseline demographic and socioeconomic indicator information (age, sex, rural status, neighborhood income quintile) and also derived a Charlson Comorbidity Score for each patient by assessing diagnostic codes recorded in hospital records in the 5 years prior to the home care admission date [13–15].

### Statistical methods

We quantified the agreement between the administrative database case definition for pilonidal sinus and diabetic foot ulcer and those collected by the centers using the Kappa statistic. For all baseline characteristics we expressed continuous variables as means and standard deviations (SD) or medians and interquartile range (IQR), and categorical variables as proportions. We used standardized differences, which reflect the mean between-group differences as a proportion of the pooled SD, to assess for differences between groups; a standardized difference greater than 10% is generally considered meaningful [16]. Unlike *t* tests and other statistical tests of hypothesis, standardized differences are not influenced by overall sample size, between-group differences in sample size, or clustering [17]. We graphically assessed the differences in groups on successful discharge from home care using Kaplan-Meier plots.

As the purpose of the outcome-based pathway is to promote coordinated care resulting in earlier wound healing, our principle analysis was a time-to-event analysis using multivariable Cox proportional hazards regression. Using this model, we derived hazard ratios (HR) and 95% confidence intervals (CI) for the study outcome, with the control group serving as the reference for all analyses. Clients were censored if they died, were admitted to hospital, were lost to follow-up (went on vacation for more than 30 days), were admitted to a long-term care facility or were not discharged by the maximum follow-up date of 30 April 2014. All models accounted for the potential clustering by center and were adjusted for potential confounders: age, sex, rural residence, neighborhood income quintile, and Charlson Comorbidity Score. We performed all hypothesis tests using a two-sided test and interpreted a *p* value < 0.05 as statistically significant. All statistical analyses were conducted with SAS for UNIX version 9.2 (SAS Institute, Cary, NC, USA).

## Results

### Sample

**Fig. 2:**
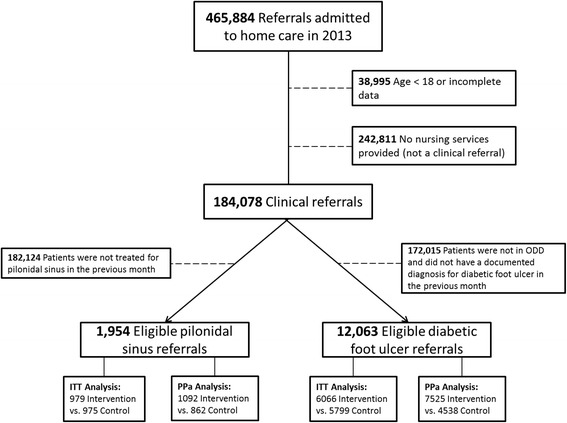
Patient inclusion and exclusion diagram

We identified 465,884 referrals to any of the 14 Ontario home care centers in 2013. Of these, 38,995 had invalid or incomplete data or were for clients under 18 years of age. We further excluded 242,811 referrals that received no nursing services as they represented a nonclinical referral, for a total of 184,078 clinical home care referrals. Among these, we identified 12,063 (6.6%) meeting the definition of diabetic foot ulcer and 1954 (1.1%) meeting the definition of pilonidal sinus, as defined in the “Methods” section (Fig. 2). Case definition and outcome variables were complete for all patients. Neighborhood income quintile was imputed to the middle category for 0.6% of patients and rurality was imputed with the most common value (urban residence) for 0.05% of patients. No other data were missing. The Kappa agreement for the case definitions was 0.20 (95% CI, 0.198 to 0.206) and 0.36 (95% CI, 0.353 to 0.362), respectively.

### Diabetic foot ulcer cohort

#### Baseline data

Patients were a median of 66 year of age (IQR, 56–76) and were mostly male (60.7%). Apart from the diagnosis of diabetes, a large proportion (45.6%) had significant additional comorbidities as indicated by a Charlson Comorbidity Score of 2 or more (Table 3). No appreciable differences in patient characteristics were observed between those in the control and intervention arms for either of the primary (ITT) or secondary analyses (PP).

**Table 3:** Baseline characteristics of home care referrals with a diabetic foot ulcer diagnosis in 2013

Characteristic	Control (*N* = 6066)	Intervention (*N* = 5997)	Combined (*N* = 12,063)	Standardized difference
Age – years				5%
Mean ± SD	64.82 ± 14.13	65.60 ± 14.21	65.21 ± 14.18	
Median (IQR)	65 (55–75)	66 (56–76)	66 (56–76)	
Female sex	2319 (38.23%)	2425 (40.44%)	4744 (39.33%)	4%
Urban status	5314 (87.60%)	5055 (84.29%)	10369 (85.96%)	9%
Neighborhood income quintile				
Low	1665 (27.45%)	1339 (22.33%)	3004 (24.90%)	12%
	1411 (23.26%)	1211 (20.19%)	2622 (21.74%)	7%
	1167 (19.24%)	1236 (20.61%)	2403 (19.92%)	3%
	940 (15.50%)	1199 (19.99%)	2139 (17.73%)	12%
High	837 (13.80%)	986 (16.44%)	1823 (15.11%)	7%
Charlson Comorbidity Score				
0–1	3211 (52.93%)	3340 (55.69%)	6551 (54.31%)	6%
2–3	1734 (28.59%)	1587 (26.46%)	3321 (27.53%)	5%
≥4	1121 (18.48%)	1070 (17.84%)	2191 (18.16%)	2%

IQR interquartile range, SD standard deviation

#### Primary analysis

**Fig. 3:**
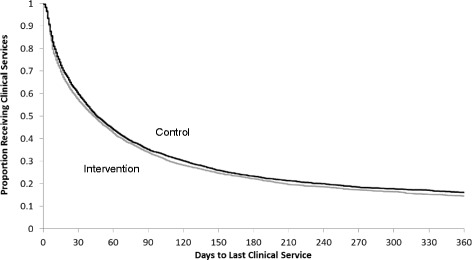
Kaplan-Meier curve of the proportion of clients who were successfully discharged from home care over the study period among the home care referrals with a diagnosis of diabetic foot ulcer (intention-to-treat (ITT) analysis)

In the ITT analysis, 72.7% (4411/6066) of patients in the control arm and 73.6% (4412/5997) of patients in the intervention arm were discharged in the follow-up period with a median time to successful discharge of 26 days (IQR 9–65) and 23 days (IQR 8–62), respectively. Results from the adjusted analysis provide no evidence to suggest a difference in the successful discharge between the two arms (HR 1.05; 95% CI 0.94 to 1.17; *p* value = 0.39) (Table 4 and Fig. 3).

**Table 4:** Outcomes analysis for home care referrals with diagnosis of diabetic foot ulcer

Analysis	Home care discharge No. successful discharges/total no. patients (%)	^a^Adjusted hazard ratio (95% CI)	*p* value
Primary analysis – ITT			
Control	4411/6066 (72.7)	Reference	
Intervention	4412/5997 (73.6)	1.05 (0.94–1.17)	0.39
Secondary analysis – PP			
Control	5495/7525 (73.0)	Reference	
Intervention	3328/4538 (73.3)	1.05 (0.94–1.16)	0.39

^a^Results from a Cox proportional hazards model adjusted for age, sex, rural residence, neighborhood income quintile, and Charlson Comorbidity Score

*ITT* intention-to-treat, *PP* per-protocol

#### Additional analyses

The results of additional analyses were consistent with the primary analyses (Table 6).

### Pilonidal sinus cohort

#### Baseline data

Patients were a median of 34 year of age (IQR 24–48) and were mostly male (67.5%). Apart from the diagnosis for pilonidal sinus, these patients were predominantly healthy as indicated by a Charlson Comorbidity Score of 0 or 1 (95%) (Table 5). No appreciable differences were observed in patient characteristics between those in the control and intervention arms for either of the primary (ITT) or secondary analyses (PP).

**Table 5:** Baseline characteristics of home care referrals with pilonidal sinus diagnosis in 2013

Characteristic	Control (*N* = 979)	Intervention (*N* = 975)	Combined (*N* = 1954)	Standardized difference
Age – years				3%
Mean ± SD	37.09 ± 15.66	37.64 ± 15.84	37.36 ± 15.75	
Median (IQR)	33 (24–48)	34 (24–49)	34 (24–48)	
Female sex	312 (31.87%)	323 (33.13%)	635 (32.50%)	3%
Urban status	879 (89.79%)	881 (90.36%)	1760 (90.07%)	2%
Neighborhood income quintile				
Low	213 (21.76%)	169 (17.33%)	382 (19.55%)	11%
	191 (19.51%)	168 (17.23%)	359 (18.37%)	6%
	206 (21.04%)	194 (19.90%)	400 (20.47%)	3%
	193 (19.71%)	253 (25.95%)	446 (22.82%)	15%
High	175 (17.88%)	186 (19.08%)	361 (18.47%)	3%
Charlson Comorbidity Score				
0–1	934 (95.40%)	929 (95.28%)	1863 (95.34%)	0%
2–3	38 (3.88%)	30 (3.08%)	68 (3.48%)	4%
≥4	7 (0.72%)	16 (1.64%)	23 (1.18%)	9%

IQR interquartile range, SD standard deviation

#### Primary analysis

**Fig. 4:**
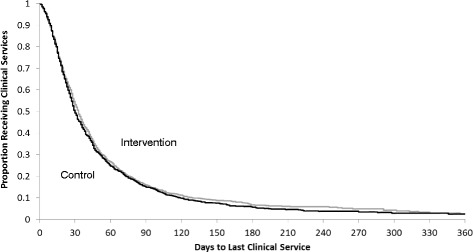
Kaplan-Meier curve of the proportion of clients who were successfully discharged from home care over the study period among the home care referrals with a diagnosis of pilonidal sinus (intention-to-treat (ITT) analysis)

In the ITT analysis, 91.0% (891/979) of patients in the control arm and 89.0% (868/975) of patients in the intervention arm were discharged in the follow-up period with a median time to successful discharge of 28 days (IQR 16 to 53) and 30 days (IQR 16 to 52), respectively. Adjusted analysis provide no evidence to suggest a difference in the successful discharge between the two arms (HR 0.96; 95% CI 0.82 to 1.12; *p* value = 0.58) (Table 6 and Fig. 4).

**Table 6:** Outcomes analysis for home care referrals with diagnosis of pilonidal sinus

Analysis	Home care discharge No. successful discharges/total no. patients (%)	^a^Adjusted hazard ratio (95% CI)	*p* value
Primary analysis – ITT			
Control	891/979 (91.0)	Reference	
Intervention	868/975 (89.0)	0.96 (0.82–1.12)	0.58
Secondary analysis – PP			
Control	981/1092 (89.9)	Reference	
Intervention	778/862 (90.3)	0.95 (0.80–1.11)	0.50

^a^Results from a Cox proportional hazards model adjusted for age, sex, rural residence, neighborhood income quintile, and Charlson Comorbidity Score

*ITT* intention-to-treat, *PP* per-protocol

#### Additional analyses

The results of additional analyses were consistent with the primary analyses (Table 4).

## Discussion

The intended objective of this pragmatic randomized controlled trial (pRCT) was to compare wound healing rates of clients treated by SPOs and CCACs randomized to the intervention arm (access to and ostensibly using outcome-based pathways), to those of clients treated by providers working with CCACs in the control group (standard practice, no outcome-based pathways). Our primary, ITT, analysis shows no evidence of a difference (either increase or decrease) in successful discharge compared to those in the standard-of-care group. Given that the trial was pragmatic (intended to answer whether or not this intervention was effective in the context of the Ontario health system) and as large as possible (including all eligible patients receiving home care for either diabetes-related ulceration or pilonidal sinus in an entire province over a period of a year) this leads to the conclusion that, as implemented, in the short term the outcome-based pathways failed to improve wound healing times in comparison with usual care approaches.

Our per-protocol analysis which corrects for contamination due to two randomized CCACs electing not to implement the intervention, and one control CCAC which chose to implement early, also showed no impact; further confirming that the intervention was not effective in achieving the intended goals even after allowing for the fact of local variability in implementation.

The ICCP initiative was a complex intervention introduced into a complex system – transformation to a new way of tracking outcomes and receiving payment in multiple independent organizations with multiple accountabilities and relationships. Complex interventions have greater scope for variation in their delivery, and so are vulnerable to one or more components not being implemented as originally intended for a variety of reasons [18]. Our parallel qualitative work suggests that the organizational commitment to the intended intervention was weak and, therefore, pathway implementation was incomplete and the pathways were actually not applied to all eligible patients. While the quantitative results reported here largely show no effect of the intervention, we determined that this was largely because the original intervention was substantially diluted in implementation and severely impacted by weak project management and incomplete engagement by key stakeholders. A key element, outcome-based reimbursement (Fig. 1), was never initiated to complement the change to an outcome-based focus and so there was a significant lack of impetus for the CCACs and SPOs to fully engage with the intervention.

The major strengths of this study are that it is a randomized trial, eliminating confounding and cancelling out imprecision in measurement; and that it is as large as it is possible to be, given that essentially all patients with the condition of interest during the period of interest, in the entire province of Ontario, were included in the study thanks to the comprehensive coverage of ICES data.

We chose a cluster randomized evaluation design in order to minimize contamination by allocating entire natural units (CCACs and their service provider partners). One view of this trial might argue that the crossover of three out of the 14 CCACs to the other arm of the trial (two from intervention to control, one from control to intervention) as well as the relatively weak implementation of the intervention in each CCAC (with poor coverage of eligible patients, and a very slow ramp-up towards a less than complete coverage) suggests that the trial itself was flawed. Using the concept of pragmatism as meaning a real-world trial, we would suggest that this result is the actual real-world performance of the intervention. Thus, far from being a failed trial, we would argue that it is an accurate evaluation of the impact of this novel intervention under real-world conditions as they currently exist in Ontario home care.

The data themselves have inherent limitations in that they are administrative data. The number of clusters is small, but this is a fixed limitation of this real-world implementation study which randomized all 14 CCACs in the province of Ontario. In terms of patient populations, the ICES definition of diabetes has been validated [12]; however, similar to other administrative definitions, likely suffers from spectrum bias where cases of less severe diabetes (generally managed in the primary-care setting) are less likely to be captured, while more severe and long-standing cases that require specialty care and hospital visits are more likely to be captured. The patients who have both diabetes and an ulcer are extremely likely to fall into the latter group as ulceration due to diabetes is a late-stage complication [19]. Similarly, for pilonidal sinus, the differential diagnosis is straightforward and involves a surgical procedure which is accurately captured on ICES data [20]; while this may not be always correct, the imprecision is washed out in both arms due to the randomization preventing bias. One other advantage of administrative data is that it allows for large, but affordable, trials. The outcomes were measured within an existing administrative data system which reduced primary data collection burden for the RCT to zero. Lastly, we only included patients with confirmation that nursing services were provided. Given the spectrum of severity of both conditions, we would assume that home care requiring nursing services within 30 days of the diagnosis of the condition would receive some management for these diseases. It is possible that some patients were receiving nursing care for other conditions entirely, and their diabetes or pilonidal sinus wounds were not being dressed by the visiting nurse, but this seems unlikely given the relative severity of these two wounds (albeit chronically for diabetes and acutely for pilonidal sinus). Even if this was a problem, because this is a randomized trial, this would only have widened the confidence interval rather than biased the point estimate of effect.

## Conclusion

Faced with the setting and results outlined above, the only responsible conclusion that can be drawn is that the intervention is not yet sufficiently proven in Ontario CCACs for widespread implementation. Significant work remains to be done to correct the implementation process such that the concept of outcome-based health care can be properly evaluated.

## Footnotes

1 Outcome pathways are the quality management tool used by the CCACs to measure whether patients were meeting expected timelines for initiation of each stage of evidence-based care and milestones towards and including wound healing.

2 Clinical pathways are multidisciplinary care tools based on evidence-based clinical practices (as against outcomes) for a specific group of patients with a predictable clinical course.
